# Allopurinol in chronic kidney disease: navigating between pathophysiology, experimental evidence, and real-world challenges

**DOI:** 10.1590/2175-8239-JBN-2026-E011en

**Published:** 2026-08-14

**Authors:** Ana Beatriz Vargas-Santos, Rosa Weiss Telles, Geraldo da Rocha Castelar-Pinheiro

**Affiliations:** 1 Universidade do Estado do Rio de Janeiro, Hospital Universitário Pedro Ernesto, Serviço de Medicina Interna, Unidade de Reumatologia, Rio de Janeiro, RJ, Brazil.; 2 Universidade Federal de Minas Gerais, Faculdade de Medicina, Departamento de Clínica Médica, Belo Horizonte, MG, Brazil.; 3 Universidade do Estado do Rio de Janeiro, Faculdade de Ciências Médicas, Departamento de Medicina Interna, Disciplina de Reumatologia, Rio de Janeiro, RJ, Brazil.

To date, the pharmacological treatment of asymptomatic hyperuricemia remains a subject of intense debate. While a deep pathophysiological understanding of the deleterious effects of urate on the endothelium and renal vasculopathy prompts us to intervene, contemporary literature presents conflicting evidence. Large randomized, controlled, multicenter clinical trials, such as the CKD-FIX^1^ and PERL^2^ studies, failed to demonstrate the benefit of this treatment in reducing the loss of glomerular filtration rate (GFR). Currently, international guidelines, such as KDIGO 2024^3^, do not recommend the routine treatment of asymptomatic hyperuricemia solely for nephroprotection.

In this issue, Santos de Souza and colleagues present a real-world experience in which the group receiving allopurinol showed significantly better renal outcomes than the untreated group^4^. This is a retrospective study based on a review of medical records conducted at a specialized center. Patients with stages 3 or 4 chronic kidney disease (CKD) and hyperuricemia, with a minimum follow-up of 24 months, were evaluated in two groups: 40 patients who received allopurinol and 40 patients who did not, with the initiation of therapy freely determined by the attending healthcare team. In summary, the treated group showed a significant improvement in GFR (from 35.06 ± 11.07 mL/min/ 1.73 m^2^ at T0 to 46.89 ± 14.57 mL/min/ 1.73 m^2^ at T3), and no patient progressed to stage 5 CKD. In contrast, the untreated group showed a significant worsening of GFR (from 40.41 ± 11.91 mL/min/1.73 m^2^ at T0 to 29.80 ± 9.76 mL/min/1.73 m^2^ at T3), with four patients pro­gressing to stage 5 CKD.

Although the favorable outcome for allopurinol is consistent with previous studies and biological data^5^, the magnitude of the effect observed in the present study raises a necessary warning regarding fundamental methodological issues. Requiring a continuous 24-month follow-up as an inclusion criterion introduces a survival bias, excluding potentially more severely ill patients from the final analysis: those who died, experienced severe adverse reactions to allopurinol, or were lost to follow-up. Furthermore, while T0 for the treated group was the evaluation immediately preceding the initiation of allopurinol, it remains unclear how T0 was defined for the comparator group. The lack of group matching through advanced statistical methods, such as the Propensity Score^6^, substantially undermines causal inference, increasing the impact of confounding by indication. Without this control, critical confounding variables—such as the etiology of CKD or the degree of proteinuria—prevent the improved performance from being categorically attributed to allopurinol. Finally, presenting GFR data exclusively as aggregate mean values at each visit, without reporting the distribution of individual changes, gives disproportionate weight to potential outliers, thereby skewing the interpretation of the results.

Although we cannot draw a definitive conclusion from this study regarding the benefit of hypouricemic therapy in the context of asymptomatic hyperuricemia and CKD, it is extremely important to highlight the wealth of data available in the literature concerning the safety of allopurinol in patients with CKD. Even though the major randomized clinical trials have failed to demonstrate nephroprotection, none has demonstrated an increased risk or renal harm associated with the drug.

Practical mastery of this safety profile is fundamental to optimizing the management of countless patients with CKD who have formal indications for allopurinol use, such as those with gout. An alarming statistic, repeated worldwide, indicates that less than half of patients with a formal indication for hypouricemic therapy are actually treated, and, among those who receive a prescription, many remain underdosed^7^.

Part of this historical undertreatment stems from excessive fear of drug toxicity and the inappropriate perpetuation of the dose equivalence table based solely on creatinine clearance (Hande et al., 1984)^8^. In recent decades, compelling evidence has redefined the safe use of allopurinol, with a focus on mitigating allopurinol hypersensitivity syndrome (AHS/DRESS)—a severe but rare idiosyncratic reaction. In this regard, the main recommendations include^9^:

1. Always start allopurinol at a low dose (up to 100 mg/day—or up to 50 mg/day in patients with advanced CKD).
2. Titrate the dose slowly (“Start low, go slow”) according to the therapeutic target:
  - In the management of gout, the universal target is to maintain serum urate below 6 mg/dL, with stricter targets (<5 mg/dL) considered for severe and tophaceous presentations.
  - In asymptomatic hyperuricemia, since phar­macological treatment is not recommended, this target remains undefined.
3. Monitor clinical and laboratory parameters, with special attention to the development of a skin rash, eosinophilia, or elevated trans­aminases, especially during the first months of treatment.
4. Consider testing for HLA-B*58:01 in populations at high epidemiological risk (e.g., individuals of Asian ancestry) before initiating treatment.
5. Pay attention to drug interactions, especially the concomitant use of loop or thiazide diuretics.

The inconsistency in the efficacy data of allopurinol as a nephroprotective strategy in asymptomatic hyperuricemia likely reflects a complex interplay of methodological aspects and biological heterogeneity^10^. Different CKD etiologies (diabetic nephropathy, hypertensive nephrosclerosis, or glomerulopathies) respond differently to urate-mediated oxidative stress and, consequently, to its control. Factors such as the duration of CKD progression at the time of intervention, the magnitude and chronicity of hyperuricemia, the follow-up duration required to capture the outcome, and the choice between fixed doses and target-guided doses are variables that still demand clear answers.

Despite all the pertinent methodological questions, the main and most valuable contribution of the study by Santos de Souza and colleagues is the legitimate and necessary call for low-cost, highly safe, and widely accessible nephroprotective measures—an indisputable priority in public health.

## Data Availability

No new data were generated or analyzed in this study.
