# Supplementary material for: Allopurinol in chronic kidney disease: navigating between pathophysiology, experimental evidence, and real-world challenges
Source: J Bras Nefrol. 2026 Aug 14;48(3):e2026E011. doi: 10.1590/2175-8239-JBN-2026-E011en (PMC13475758; doi:10.1590/2175-8239-JBN-2026-E011en)
Supplement: Translation

# Translation: Alopurinol na doença renal crônica: navegando entre a fisiopatologia, evidências experimentais e desafios da vida real

**DOI:** 10.1590/2175-8239-JBN-2026-E011pt

**Authors:** Ana Beatriz Vargas-Santos, Rosa Weiss Telles, Geraldo da Rocha Castelar-Pinheiro

**Affiliations:** 1 Universidade do Estado do Rio de Janeiro, Hospital Universitário Pedro Ernesto, Serviço de Medicina Interna, Unidade de Reumatologia, Rio de Janeiro, RJ, Brasil.; 2 Universidade Federal de Minas Gerais, Faculdade de Medicina, Departamento de Clínica Médica, Belo Horizonte, MG, Brasil.; 3 Universidade do Estado do Rio de Janeiro, Faculdade de Ciências Médicas, Departamento de Medicina Interna, Disciplina de Reumatologia, Rio de Janeiro, RJ, Brasil.

Até o momento, o tratamento farmacológico da hiperuricemia assintomática persiste sob intenso debate. Enquanto o profundo conhecimento fisiopatológico sobre os efeitos deletérios do urato no endotélio e na vasculopatia renal nos induz a querer intervir, a literatura contemporânea apresenta evidências conflitantes. Grandes ensaios clínicos randomizados, controlados e multicêntricos, como os estudos CKD-FIX^1^ e PERL^2^, falharam em demonstrar benefícios desse tratamento na redução da perda da taxa de filtração glomerular (TFG). Atualmente, diretrizes internacionais, como o KDIGO 2024^3^, não recomendam o tratamento rotineiro da hiperuricemia assintomática visando exclusivamente à nefroproteção.

Nesta edição, Santos de Souza e colaboradores apresentam uma experiência de vida real em que o grupo que recebeu o alopurinol evoluiu com desfechos renais significativamente melhores do que o grupo não tratado^4^. Trata-se de um estudo retrospectivo, baseado em revisão de prontuários, realizado em um centro especializado. Pacientes com doença renal crônica (DRC) nos estágios 3 e 4 e hiperuricemia, com seguimento mínimo de 24 meses, foram avaliados em dois grupos: 40 pacientes que receberam alopurinol e 40 pacientes que não o receberam, com a instituição da terapêutica sendo definida de forma livre pela equipe assistente. Em resumo, o grupo tratado apresentou melhora significativa da TFG (de 35,06 ± 11,07 mL/min/1,73 m^2^ em T0 para 46,89 ± 14,57 mL/min/1,73 m^2^ em T3), não tendo nenhum paciente progredido para DRC estágio 5. Em contrapartida, o grupo não tratado evidenciou piora significativa da TFG (de 40,41 ± 11,91 mL/min/1,73 m^2^ em T0 para 29,80 ± 9,76 mL/min/1,73 m^2^ em T3), com 4 pacientes evoluindo para DRC estágio 5.

Embora o desfecho favorável ao alopurinol seja consistente com estudos prévios e dados biológicos^5^, a magnitude do efeito apresentada no presente estudo desperta um alerta necessário quanto a questões metodológicas fundamentais. Exigir o acompanhamento contínuo por 24 meses como critério de inclusão introduz um viés de sobrevida, excluindo da análise final os pacientes potencialmente mais graves: aqueles que evoluíram para óbito, apresentaram reações adversas graves ao alopurinol ou foram perdidos durante o seguimento. Além disso, enquanto o T0 do grupo tratado correspondeu à avaliação imediatamente anterior ao início do alopurinol, permanece incerto como o T0 foi definido para o grupo comparador. A ausência de pareamento dos grupos por métodos estatísticos avançados, como o Escore de Propensão^6^, prejudica substancialmente a inferência de causalidade, aumentando o impacto do viés de indicação do medicamento (*confounding by indication*). Sem esse controle, variáveis de confusão críticas — como a etiologia da DRC ou o grau de proteinúria — impedem que o melhor desempenho seja categoricamente atribuído ao alopurinol. Por fim, a apresentação dos dados de TFG exclusivamente como médias agregadas em cada visita, sem reportar a distribuição do delta individual, confere um peso desproporcional a possíveis *outliers*, enviesando a interpretação dos resultados.

Ainda que não possamos extrair deste estudo uma conclusão definitiva sobre o benefício do uso de hipouricemiantes no contexto da hiperuricemia assintomática e da DRC, é extremamente importante ressaltar a riqueza de dados disponíveis na literatura sobre a segurança do alopurinol em pacientes com DRC. Embora os principais ensaios clínicos randomizados tenham falhado em demonstrar nefroproteção, nenhum indicou incremento do risco ou dano renal associado ao fármaco.

O domínio prático dessa segurança é fundamental para otimizar o manejo de inúmeros pacientes com DRC que possuem indicações formais para o uso de alopurinol, como aqueles com gota. Um dado alarmante e repetido globalmente indica que menos da metade dos pacientes com indicação formal de hipouricemiantes são, de fato, tratados e, dentre os que recebem a prescrição, muitos permanecem em subdose^7^.

Parte desse subtratamento histórico decorre do receio excessivo quanto à toxicidade do fármaco e da perpetuação inadequada da tabela de equivalência de dose baseada estritamente na depuração da creatinina (Hande et al., 1984)^8^. Nas últimas décadas, evidências contundentes redefiniram o uso seguro do alopurinol, com foco na mitigação da síndrome de hipersensibilidade ao alopurinol (AHS/DRESS) — uma reação idiossincrática grave, porém rara. Nesse sentido, as principais recomendações incluem^9^:

1. Iniciar o alopurinol sempre em dose baixa (até 100 mg/dia – ou até 50 mg/dia em pacientes com DRC avançada).
2. Titular a dose lentamente (*“Start low, go slow”*) conforme o alvo terapêutico:
  - No manejo da gota, o alvo universal é manter a uricemia abaixo de 6 mg/dL, considerando-se alvos mais estritos (<5 mg/dL) para quadros graves e tofáceos.
  - Na hiperuricemia assintomática, como o tratamento farmacológico não é reco­mendado, esse alvo permanece indefinido.
3. Monitorar clínica e laboratorialmente, com atenção especial ao desenvolvimento de rash cutâneo, eosinofilia ou elevação de transaminases, principalmente nos primeiros meses do tratamento.
4. Considerar a pesquisa do HLA-B*58:01 em populações de alto risco epidemiológico (ex.: ascendência asiática) antes de iniciar o medicamento.
5. Ter atenção às interações medicamentosas, especialmente uso concomitante de diuréticos de alça ou tiazídicos.

A inconsistência nos dados de eficácia do alopurinol como estratégia de nefroproteção na hiperuricemia assintomática provavelmente reflete uma complexa interação entre aspectos metodológicos e heterogeneidade biológica^10^. Diferentes etiologias de DRC (nefropatia diabética, nefroesclerose hipertensiva ou glomerulopatias) respondem de forma distinta ao estresse oxidativo mediado pelo urato e, consequentemente, ao seu controle. Fatores como o tempo de evolução da DRC no momento da intervenção, a magnitude e a cronicidade da hiperuricemia, o tempo de seguimento necessário para captar o desfecho e a escolha entre doses fixas *versus* doses guiadas por metas são variáveis que ainda demandam respostas claras.

A despeito de todos os questionamentos metodológicos pertinentes, a principal e mais valiosa contribuição do estudo de Santos de Souza e colaboradores é o legítimo e necessário clamor por medidas nefroprotetoras de baixo custo, alta segurança e amplo acesso—uma prioridade indiscutível em saúde pública.

## Data Availability

Nenhum dado novo foi gerado ou analisado neste estudo.
